# Supplementary material for: Isolation and Characterization of Euglena gracilis-Associated Bacteria, Enterobacter sp. CA3 and Emticicia sp. CN5, Capable of Promoting the Growth and Paramylon Production of E. gracilis under Mixotrophic Cultivation
Source: Microorganisms. 2021 Jul 13;9(7):1496. doi: 10.3390/microorganisms9071496 (PMC8303684; doi:10.3390/microorganisms9071496)
Supplement: Supplementary file 1 [file microorganisms-09-01496-s001.zip › microorganisms-1252066-supplementary.pdf]

Supplementary Materials

Table S1 List of bacterial strains isolated in this study.

Name	Identification *	
	Closest strain	Similarity, bp (%)
CA1	*Microbacterium neimengense* 7087^T	814/822 (99.0%)
CA2	*Microbacterium neimengense* 7087^T	806/812 (99.3%)
CA3	*Enterobacter kobei strain* JCM 8580^T	804/822 (97.8%)
CA4	*Kaistia algarum* LYH11^T	751/791 (94.9%)
CA6	*Achromobacter insolitus* LMG 6003^T	816/819 (99.6%)
CA7	*Microbacterium neimengense* 7087^T	805/812 (99.1%)
CA8	*Achromobacter insolitus* LMG 6003^T	802/816 (98.3%)
CA9	*Achromobacter insolitus* LMG 6003^T	814/827 (98.4%)
CN1	*Pedobacter boryungensis* BR-9^T	665/694 (95.8%)
CN2	*Phenylobacterium lituiforme* FaiI3^T	794/809 (98.1%)
CN3	*Sphingomonas sanguinis* NBRC 13937^T	779/815 (95.6%)
CN4	*Achromobacter insolitus* LMG 6003^T	816/818 (99.6%)
CN5	*Emticicia fontis* IMCC1731^T	788/823 (95.7%)
CN6	*Elizabethkingia miricola* W3-B1^T	804/808 (99.5%)
CN7	*Enterobacter kobei strain* CIP 105566^T	800/822 (97.3%)
CN8	*Herminiimonas contaminans* CCUG 53591^T	818/821 (99.6%)
CN9	*Sediminibacterium goheungense* HME7863^T	799/803 (99.5%)
CN10	*Pedobacter boryungensis* BR-9^T	804/818 (98.3%)
CN11	*Erythrobacter xanthus* SM1501^T	773/810 (95.4%)
CN12	*Pedobacter boryungensis* BR-9^T	797/818 (97.4%)
CN13	*Sediminibacterium roseum* SYL130^T	804/820 (98.0%)
CN14	*Polaromonas eurypsychrophila* B717-2^T	790/812 (97.3%)
CN15	*Sediminibacterium goheungense* HME7863^T	788/801 (98.4%)

*Partial 16S rRNA genes were amplified by PCR using primers 10F (5′-GTTTGATCCTGGCTCA-3′) and 810R (5′-TACCAGGGTATCTAATCC-3′). The closest strains were identified based on 16S rRNA gene-sequence similarities with type strain sequences, which were determined by performing BLAST searches against NCBI GenBank.

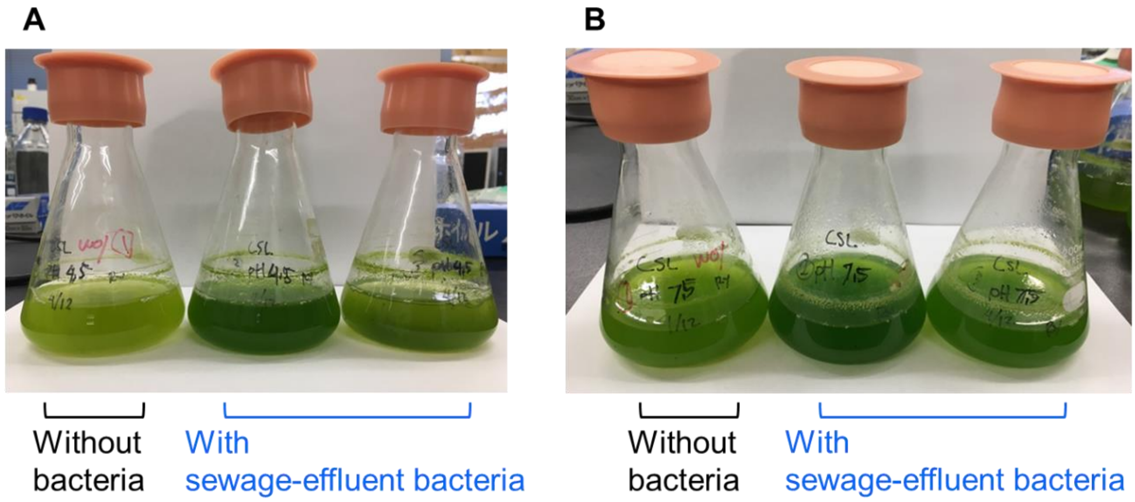

Figure S1. Images of *Euglena gracilis* cultures in C-NH_4 medium with 0.5 g L^{-1} corn steep liquor (CSL) at pH 4.5 (A) or pH 7.5 (B) and with or without sewage-effluent bacteria for 10 d. The final biomass concentrations of *E. gracilis* grown in C-NH_4 with 0.5 g L^{-1} CSL with or without effluent bacteria at pH 4.5 were 349.3 ± 15.9 mg L^{-1} and 201.4 ± 8.5 mg L^{-1}, respectively, and at pH 7.5 were 428.6 ± 47.1 mg L^{-1} and 279.3 ± 23.1 mg L^{-1}, respectively.

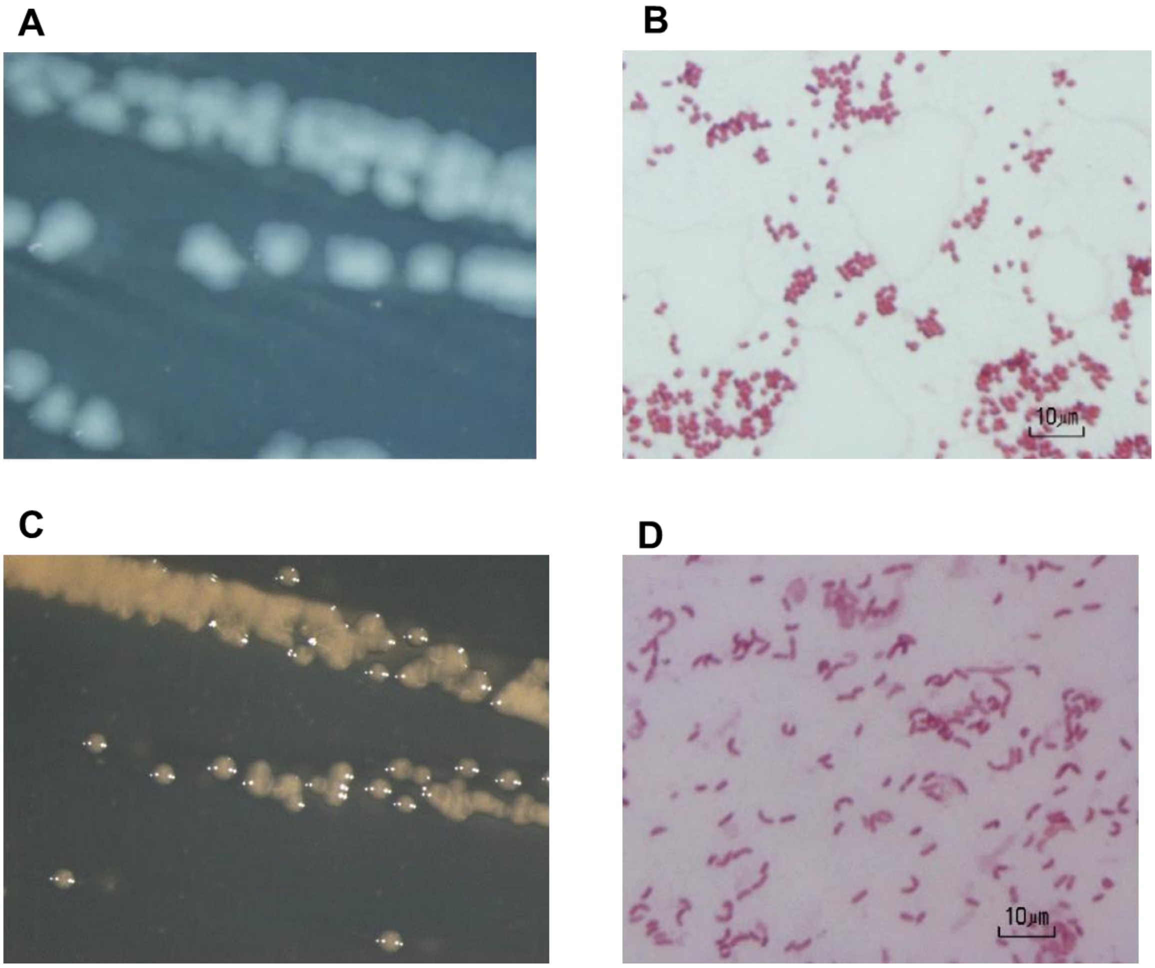

Figure S2. Images of *Enterobacter* sp. CA3 (acidophilic bacterium) colonies on R2A agar (A) and a light microscope image after Gram staining (B) and images of *Emticicia* sp. CN5 (neutrophilic bacterium) colonies on R2A agar (C) and a light microscope image after Gram staining (D).
